# 0041. The effect of low-dose hydrocortisone after severe burn injury: a microarray longitudinal study

**DOI:** 10.1186/2197-425X-2-S1-O10

**Published:** 2014-09-26

**Authors:** J Textoris, J Plassais, M-A Cazalis, F Venet, T Rimmele, G Monneret, A Pachot, S Tissot

**Affiliations:** BioMérieux, Joint Research Unit Sepsis, Lyon, France; Hospices Civils de Lyon, Burn ICU, Lyon, France; Hospices Civils de Lyon, Immunology Lab, Lyon, France

## Introduction

The systemic effect of inflammatory mediators release after severe burn is a severe cardiovascular dysfunction called burn shock. Patients need vasopressor infusion to maintain adequate delivery of oxygen but it could have deleterious effects on skin perfusion and worsen the burn depth. This shock could result from the interplay of the initial hypovolemia and the release of multiple inflammatory mediators. It has been shown that a low-dose of hydrocortisone could reduce the shock duration but the mechanisms involved remain unclear. In this study, we investigated the systemic genomic response after severe burn injuries and determine whether patterns of gene expression could be associated with low-dose of glucocorticoids.

## Objectives

In this study, we investigated the systemic genomic response after severe burn injuries and determine whether patterns of gene expression could be associated with low-dose of glucocorticoids.

## Methods

Thirty burn patients with over 30% of total body surface area were enrolled into a randomized double-blind clinical study. 15 patients were treated with low-dose of hydrocortisone and 15 patients were treated with placebo. Whole blood samples were collected after shock onset (S1) before any treatment, one day after treatment beginning (S2) and 120h and 168h after the burn injury (S3/S4). Moreover blood samples of 13 healthy volunteers were collected. Pangenomic expression was evaluated with Affymetrix HG-U133plus 2.0 microarrays. Moderated t-tests and F-test were used to compare burn patients to controls and then gene expression profiles between the 2 groups (B-H correction, p< 0.05).

## Results

Severe burn injury induced the deregulation of a considerable number of genes (n>2200 at S1) in comparison with controls with an increased number of deregulated genes over time. Within burn patients, more than 300 genes were deregulated by hydrocortisone over time. The treatment had a rapid effect on gene expression, 339 and 627 genes were differentially expressed at S2 and S3 respectively. However the number of these genes decreased drastically at S4 (only 24 genes significant). The genes identified at S2 were mostly related to the decrease of growth, development and quantity of leukocytes but these biological processes were not found significant at S3 indicating that the action of glucocorticoid in the response to burn injury is short-lived and time dependent.

## Conclusions

This study is an informative overview of the genomic responses after burn injuries. More importantly it is the first study providing information about mechanism involved in glucocorticoid's reduced shock duration after burn.

